# P-1136. Assessing Quantitative PCR and Standard Microbiologic Cultures for Endoscopic Surveillance: A Disposable Bronchoscope Contamination Model

**DOI:** 10.1093/ofid/ofaf695.1330

**Published:** 2026-01-11

**Authors:** Nouf K Almaghlouth, Simran Singh, Mohamed Yassin

**Affiliations:** University of Pittsburgh Medical Center , Pittsburgh, PA; University of Pittsburgh, Pittsburgh, Pennsylvania; University of Pittsburgh, Pittsburgh, Pennsylvania

## Abstract

**Background:**

Bronchoscopy is a cornerstone of both diagnostic and therapeutic interventions, with approximately half a million procedures performed annually in the United States. Despite its critical role in patient care, bronchoscopy is associated with significant infection risks stemming from endogenous patient flora and exogenous contamination of the bronchoscope. Several outbreaks of infection linked to bronchoscopy have been reported, highlighting the need for robust surveillance systems to detect and prevent bronchoscope contamination. This study aims to assess the comparative efficacy of standard microbiology cultures versus quantitative PCR testing in detecting bronchoscopic contamination.

**Methods:**

A standardized strain of *Pseudomonas aeruginosa* (ATCC 27853) was cultured in LB broth to achieve a concentration of 10⁵ CFU/mL. Single-use bronchoscopes (SUBs) were inoculated with 20 mL of the bacterial solution and artificial bronchoalveolar lavage fluid (simulated saliva, BZ305; pH 7.2). The inoculated SUBs were incubated for 2 hours, followed by a 20 mL flush with sterile water. The devices then underwent high-level disinfection (HLD). Pre- and post-HLD flush samples were collected and analyzed using both standard microbiologic cultures and quantitative PCR (qPCR). DNA was extracted following a standard protocol, and qPCR detection was performed using validated primers and probes, alongside a standard curve and appropriate negative and positive controls to assess assay efficiency.

**Results:**

Four unique SUBs were tested. All pre-HLD samples were positive by both culture and qPCR. Post-HLD, all cultures were negative, while qPCR remained positive, with average cycle threshold (Ct) values of 19 pre-HLD and 32 post-HLD, reflecting a 4-log reduction consistent with the established standard curve.

**Conclusion:**

This contamination model demonstrates the utility of qPCR for bronchoscope surveillance. While standard cultures detect viable organisms, qPCR offers the advantage of quantifying residual DNA, providing a measure of disinfection efficacy. Establishing Ct cutoff values is essential for interpreting qPCR results post-HLD. The integration of both cultures and qPCR could strengthen bronchoscope contamination monitoring protocols.

**Disclosures:**

All Authors: No reported disclosures

